# The Design of Experiment as a Tool to Model Plant Trace-Metal Bioindication Abilities

**DOI:** 10.3390/molecules27061844

**Published:** 2022-03-11

**Authors:** Mirko Salinitro, Alessandro Zappi, Sonia Casolari, Marcello Locatelli, Annalisa Tassoni, Dora Melucci

**Affiliations:** 1 Department of Biological Geological and Environmental Sciences, University of Bologna, 40126 Bologna, Italy; mirko.salinitro2@unibo.it (M.S.); annalisa.tassoni2@unibo.it (A.T.); 2 Department of Chemistry “G. Ciamician”, University of Bologna, 40126 Bologna, Italy; sonia.casolari@unibo.it (S.C.); dora.melucci@unibo.it (D.M.); 3 Department of Pharmacy, University “G. D’Annunzio” of Chieti-Pescara, 66100 Chieti, Italy; marcello.locatelli@unich.it

**Keywords:** bioindicator plants, DoE, metal pollution, metal uptake, multivariate analysis

## Abstract

Bioindicator plants are species that have the capacity to linearly uptake some elements (metal and metalloids) from the growing substrate, thus reflecting their concentration in the soil. Many factors can influence the uptake of these elements by plants, among which is the simultaneous presence of several metals, a common situation in contaminated or natural soils. A novel approach that can be used to validate the bioindication ability of a species growing on a polymetallic substrate is the design of experiment (DoE) approach. The aim of the present study was to apply the DoE in full factorial mode to model the Cu, Cd, Pb, Zn, and Cr bioindication capacity of *Polygonum aviculare*, used as the model plant. The results showed that *P. aviculare* has the ability to bioindicate Cd and Cr with a linear uptake (from 0.35 to 6.66, and 0.1 to 3.4 mg kg^−1^, respectively) unaffected by the presence of other metals. Conversely, the uptake of Pb, Cu, and Zn is strongly influenced by the presence of all the studied metals, making their concentration in the plant shoot not proportional to that of the soil. In conclusion, these preliminary results confirmed that the DoE can be used to predict the bioindicator abilities of a plant for several elements at the same time and to evaluate the interactions that can be established between variables in the growing medium and in the plant itself. However, more studies including other plant species are needed to confirm the effectiveness of this method.

## 1. Introduction

The ability of plants to absorb trace metals from soils is a topic of great interest for biologists [1], because, depending on their accumulation capacity, plants could be used for bioindication [2,3] or phytoremediation purposes [4,5]. The bioindication approach uses plants as an analytical tool to monitor the presence of trace metals in the soil. Conversely, phytoremediation aims to remove the trace metals from the environment, taking advantage of plants’ capacity to absorb and transfer them from soil to their aerial parts [6]. For bioindication purposes, indicator plants are used. Their essential characteristic is the linear uptake and accumulation of certain elements from the soil, thus reflecting the actual concentration in the soil itself [7]. The bioindication approach has already been applied in the urban environment to monitor soil trace metal pollution using various weed species [2]. It is in fact well established that some trace metals, including As, Cr, Cu, Hg, Ni, Pb, Zn can have a deleterious effect on human health, thus their presence must be kept under control in densely populated areas, such as cities [8]. *Taraxacum officinale* has been widely studied for its biomonitoring capacity of several elements, including Cd, Cu, Zn, and As [9,10]. *Cichorium intybus* L. has been identified as an effective plant for the bioindication of cadmium [10]. *Achillea wilhelmsii* and *Cardaria draba*, have been reported as useful plants for bioindication as well as the phyto-stabilization of traffic-related trace metal pollutants [11,12]. Moreover, Fe, Al, Cr, Ni, Sr, V, and Zn levels in *Plantago major* were correlated to that of soil, confirming the bioindication ability of this species [9]. Finally, a recent study that involved the *Polygonum aviculare* and *Poa annua*, confirmed that these species were particularly efficient for Ni biomonitoring in urban soils [13]. Indeed, these perennial weeds are gaining attention for bioindication purposes because of their widespread presence in several habitats, including polluted ones (i.e., urban habitats, wastelands, etc.).

Since urban soils are enriched in several anthropic trace metals, some plants have also evolved hyper-accumulation traits. It is the case of the annual plant *Solanum nigrum* that has been reported to accumulate more than 100 mg/kg of Cd under both laboratory and natural soil conditions [14]. Similarly, the common knotgrass (*Polygonum aviculare* L.), was reported for its capacity to accumulate more than 10 mg/kg of Hg when growing in industrial polluted sites [15].

However, despite the capacity of common urban weeds to bioindicate or accumulate trace metals, these properties are poorly investigated. The main reason resides in the complex interactions between the soil variables (elements interactions, pH, organic matter, etc.) that often make impossible the observation of one single variable’s effect. The novelty of this study resides in the use of the design of experiment (DoE) method to determine how the different soil variables interact with each other in order to model the reaction of the studied plant. This innovative approach may be of great help in speeding up the process of discovery and validation of new indicator species.

DoE is a chemometric method that allows the building of a mathematical model describing the influence of some factors or variables (independent variables) on one or more responses (dependent variables). It is commonly used in chemistry to optimize, for example, the conditions of a reaction to maximize its yield [16] or the analytical conditions to maximize a chromatographic separation [17]. However, DoE has been widely used in many other research fields, such as engineering [18], medicine [19], and psychology [20]. The advantage of DoE is that, besides the mathematical formalization of the final model, it allows the effect of several variables to be studied at the same time and, most of all, their interactions. The accumulation of one metal, indeed, may be influenced by the presence of other metals in the growing medium. However, such interaction may be very difficult to study in a natural environment and must be evaluated in a controlled setting. Previous studies have already dealt with multi-element accumulation by plants [21,22,23]; however, they did not consider the effect of a poly-metallic growing medium on plant development [22,23] nor did they consider these relations in light of the mathematical formalism of DoE [21].

The present study aimed to apply DoE to the modeling of the trace-metal bioindication abilities of *P. aviculare*. In particular, we focused on investigating the interactions between Cu, Cd, Pb Zn, and Cr when simultaneously present in a polymetallic growing medium, and how the presence of one element can enhance or suppress the bioaccumulation of the others. In addition, the study aimed to validate the use of DoE as a feasible approach to test the performances of new indicator species, using *P. aviculare* as a model plant. Previous studies [13] have already proved that *P. aviculare* can accumulate Ni at a level comparable to that of soil (thus being a monitoring species); however, data about the above-cited elements are lacking. The modeling was carried out by growing the plants in hydroponic conditions, in fact, it has been reported [23] that the bioavailability and consequent bioaccumulation of trace metals are strongly influenced by soil characteristics, such as pH, composition, and granulometry. By using the hydroponic approach, the only source of variability left (except for the biology of plants themselves) was the concentration of the studied metals, making clearer the interactions between the studied trace metals and the consequent formulation of models.

## 2. Materials and Methods

### 2.1. Plants Cultivation

*P. aviculare* seeds were germinated in Ø 6 cm pots filled with coarse quartz sand. After germination, pots were placed in saucers filled with 250 mL half-strength Hoagland’s solution [24], in order to cover 1/3 of the pot height. The Hoagland’s solution had the following composition: KNO_3_ (2 mM); Ca(NO_3_)_2_•4H_2_O (2 mM); MgSO_4_•7H_2_O (0.5 mM); NH_4_NO_3_ (0.5 mM); KH_2_PO_4_ (0.25 mM); KCl (50 μM); Fe(Na_2_)-EDTA (40 μM); H_3_BO_3_ (25 μM); ZnSO_4_•7H_2_O (2 μM); MnCl_4_•4H_2_O (2 μM); CuSO_4_•5H_2_O (0.5 μM); CoCl_2_•6H_2_O (0.15 μM); (NH_4_)_6_Mo_7_O_24_•4H_2_O (0.075 μM); pH was adjusted to 5.8–6 with KOH. All plants were cultivated for 1 week in standard half-strength Hoagland’s solution to allow the establishment of seedlings. For each treatment, 3 pots were independently cultivated, each one containing three to five plants. After this first week, to the standard medium (already containing 0.5 μM Cu and 2 μM Zn), Cr(NO_3_)_3_•9H₂O, Cd(NO_3_)_2_•4H_2_O and Pb-EDTA were added as sources of Cr, Cd, and Pb, respectively, according to the concentrations chosen for the DoE (summarized in Table 1). Metal levels were chosen according to a previous study carried out by our group [13] which analyzed several urban and countryside soils. The analyzed urban soils (from Bologna and Milan, Italy) showed on average, Cd, Cr, and Pb concentrations reported as the “central” level of the DoE in the present study. Countryside soils were collected in natural areas near the above-cited cities, and their average Cd, Cr, and Pb concentrations are represented by the “low” level of the DoE in the present study. The “high” level of the DoE represents heavily polluted soils, with concentrations being twice those of “central” level. The summary of all the performed experiments and metal-concentrations levels are reported in Appendix A (Table A1).

All solutions were renewed every three days in order to maintain constant nutrient and metal concentrations for the entire experimental duration. All chemicals were purchased by Sigma Aldrich (Merck, Darmstadt, Germany). Plants were grown in a growth chamber at a temperature of 20 °C and a photoperiod of 16/8 h light/dark. Artificial light was provided employing TLED Growing 42 W LED lights (Secret Jardin, Manage, Belgium).

### 2.2. Sample Preparation and Analysis

Plants were harvested after 4 weeks from germination (1 week of establishment + 3 weeks of treatment), then dried in a ventilated oven at 60 °C for 24 h until constant weight. Plants were ground with an A11 basic analytical mill (IKA, Staufen, Germany) to obtain a fine powder for metals analysis. A portion of 100 mg of powder was put in a ceramic capsule and 1 mL of HNO_3_ 0.5 M was added; capsules were then put in a muffle furnace at 500 °C for 5 h. This procedure destroys and removes the organic matter that evaporates as H_2_O and CO_2_ in the furnace until only ashes are left. Ashes were dissolved in 4 mL of HNO_3_ 0.5 M, and the solution was analyzed by atomic absorption spectrometry (AAS) for their trace element content using a Perkin Elmer AAnalyst 400 (Perkin Elmer, Waltham, MA, USA), controlled by the software WinLab 32 by Perkin Elmer.

For the quantification of each of the five analyzed metals (Cd, Cr, Cu, Pb, Zn) a calibration line was computed. Standards for the calibration curves were purchased from Merck (Darmstadt, Germany). The ranges of the standard samples were chosen considering the linear range indicated by the software WinLab 32 for each metal. Therefore, for each metal, three standards were prepared, with the following concentrations: for Cd, 2–4–6 μg L^−1^; for Cr, 2–10–25 μg L^−1^; for Cu, 2.5–10–25 μg L^−1^; for Pb, 5–20–40 μg L^−1^; for Zn, 0.1–0.5–0.8 mg L^−1^.

The analytical signal used for quantification was the AAS-peak area, verifying at each measurement that the peak height never exceeded 0.6 AU, in order to keep the absorbance signal inside the linear range. Before each analysis, a blank sample was analyzed and its peak area was subtracted from that of the corresponding sample. The limit of detection (LoD) was computed for each calibration line, verifying that it never exceeded the lowest standard concentration. Three replicates of each standard and each plant sample were analyzed. The injected volume was 20 μL for each analysis. If a sample peak exceeded the linear range, its solution was properly diluted to bring it back into the calibration range. In these cases, the dilution factor was taken into account for the final calculation of the metal concentration in the plant (expressed in mg kg^−1^). All the experimental results are reported in Table A1.

For the analysis of Cd, Cr, and Pb by AAS, some matrix modifiers are necessary. In particular, Mg(NO_3_)_2_ (Perkin Elmer) was used for Cd and Cr, PdCl_2_ (Fluka, Honeywell, Morris Planes, NJ, USA) for Cd, and NH_4_H_2_PO_4_ (Sigma Aldrich) for Pb. Cu and Zn do not require modifiers. A solution containing all the modifiers was added to each plant sample, the concentrations of the modifiers were the following: 200 mg L^−1^ for Mg(NO_3_)_2_; 2.3 mg L^−1^ for PdCl_2_; 4 mg L^−1^ for NH_4_H_2_PO_4_. It was verified that the analysis of each metal was not influenced by the presence of an unnecessary modifier.

To validate the accuracy of measurements, for each metal, five plant samples were spiked with a known quantity (1000 ppm) of trace metal prior to incineration. The recovers were evaluated, and it was verified that these never exceeded the limits 90–110%, evaluating possible losses or increases during the incineration process. Moreover, to assure instrumental stability, quality control solutions (same concentrations of standards) were included in the analysis every 10 plant samples. The accepted recovery rate of control solution was fixed within the narrower range than above of 95–105%. If a value was found outside this range, the instrument calibration line was recomputed.

### 2.3. Design of Experiment

The name “design of experiment” (DoE) [25] covers several chemometric methods aimed at finding the best combination of dependent and independent variables to optimize a regression problem. A DoE starts by defining the independent variables (*x*) that will most influence the problems, which are called factors, and the dependent variables (*y*) of interest, called responses. In the present work, the factors are the metal concentrations added to the plants’ growing medium and the responses are the metal concentrations collected by the plants. To perform a DoE, a proper number of levels for each factor must be chosen, the minimum being two. If two, only a minimum and a maximum value for the factor are considered. A third level is, in general, the central value between the first two. To maximize the comparability between factors in the following computations, the minimum level is indicated by −1, the maximum by +1, and the central level by 0. The factors’ levels are the second important choice to perform for a proper DoE: these depend, obviously, on the problem and must be selected in an interval that can fully represent the variability of the factor in the real problem under examination. Once decided the number of factors (*F*) and the levels, assuming that the same number of levels (*L*) is used for all factors, the total number of experiments (*N*), without considering repetitions is given by Equation (1):

(1) $$N=L^{F}$$

If all the *N* experiments are performed, the DoE is called full-factorial design. The *N* experiments are performed taking into account all the combinations of the factors’ levels.

The advantage of DoE is that the region of the experimental space (the region spanned by minima and maxima levels of the factors) not explored by the experiments can be mathematically evaluated. Indeed, registering the response(s) for each experiment, a model can be calculated for each response in the form (Equation (2)):

(2) $$y=b_{0}+\underset{i}{\sum }b_{i}x_{i}+\underset{i,j}{\sum }b_{ij}x_{i}x_{j}+\underset{i}{\sum }b_{ii}x_{ii}^{2}$$

In Equation (2), *y* is the response, *b* are the regression coefficients, and *x* the factors. *b*_0_ is the intercept of the model, the first summation evaluates the contribution to the response of each factor by itself (*b_i_* being the regression coefficients corresponding to each *i*-th variable). The second summation evaluates, instead, the contribution of the binary interactions [26] between the factors to the response (*b_ij_* being the regression coefficient of the interaction between variables *i* and *j*). Therefore, it is an index of how two variables can be influenced by each other. The third summation evaluates the squared contributions of each factor (*b_ii_* being the corresponding regression coefficients), and it can be calculated only if three or more levels for that factor are considered in the DoE.

From Equation (2), two important conclusions about the problem can be drawn [25]. The first one concerns the variable importance for the problem: to each regression coefficient, a *p*-value can be associated; if such a *p*-value is significant (i.e., lower than the chosen significance level α, that is generally 0.05), the corresponding factor (or interaction or squared effect) is also significant. It means that a significant variable would probably be important to describe the variation of the response. If instead, the *p*-value is not significant, the corresponding factor can be considered not significant for the response variation. The second conclusion concerns the response variability. Indeed, by substituting the proper factor level into Equation (2), all the response values and the corresponding standard deviations for the entire experimental space can be calculated. The response values are reported in a graph called “response plot”, in which the abscissa and ordinate axes are two (significant) factors, and the values on the Cartesian plane are the calculated response. On the response plot, the iso-response curves are shown, which are the regions in which the response reaches the same calculated value. The semiamplitude of the confidence interval can also be visually evaluated in a graph similar to the response plot. By comparing the response plot and the corresponding semiamplitude of the confidence intervals plot (in the following, always using a significance level of 0.05), an overview about the confidence intervals of the recalculated responses can be drawn. In this way, the general behavior of the response can be evaluated in simple two-dimension plots.

All computations for the DoE were carried out by the software CAT [27], based on the R 3.1.2 environment (R Core Team, Vienna, Austria).

## 3. Results and Discussion

The design of experiment in the full factorial mode indicated the execution of 27 experiments of plant growth. Indeed, three factors were varied (the concentrations of Cd, Pb, and Cr) over three levels (−1, “0”, +1). In the present work, the “central” levels do not correspond to the mathematical center (0) between “high” (+1) and “low” (−1) levels (Table 1). Therefore, the “central” levels assumed the values −0.08, −0.07, and −0.18 for Cd, Pb, and Cr, respectively (Table A1). As already stated, the concentrations of Cu and Zn were left constant (0.5 and 2 μM, respectively). For each of the 27 DoE experiments, three pot replicates were independently cultivated, each one containing 3–5 plants, resulting in 81 total analyzed plant samples (Table A1).

After the determination of shoot concentrations of Cd, Cr, Cu, Pb, and Zn by AAS, it was possible to compute a model for each metal concentration, used as responses, such as the one shown in Equation (2). The use of three levels for each factor also allowed the evaluation of the squared effects of each factor, besides that of each factor by itself and the binary interactions in the uptake of all metals. Table 2 shows the coefficients estimated for each model. Bold values in Table 2 indicate statistically significant coefficients, i.e., whose *p*-value is lower than the significance level (α) 0.05. Such coefficients correspond to factors that may influence the response variation.

### 3.1. Cadmium

The concentration range obtained for Cd varied from 0.34 to 6.92 mg kg^−1^ (Table A1). From Table 2, it can be seen that the significant factors in the Cd model are *b*_1_ (*p* < 0.001) and the interaction *b*_13_ (*p* = 0.0118). These correspond to the factor “Cadmium” and its interaction with “Chromium”. The coefficient of *b*_1_ is positive, which means that, at least in the explored experimental space, the more Cd is added to the growth medium, the more it is absorbed by *P. aviculare*.

**Figure 1:**
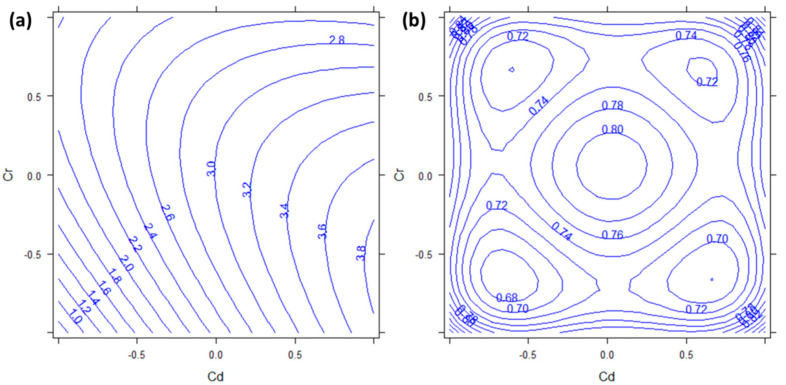
(**a**) Response surface and (**b**) semiamplitude of the 95% confidence interval for Cd response. Factors Cd and Cr are reported in abscissa and ordinate respectively. Pb has been kept constant to the 0 level.

Cd uptake behavior calculated by the DoE model is shown in the response surface reported in Figure 1. The variables reported in Figure 1 are the factors “Cd” (in abscissa) and “Cr” (in ordinate). The lines reported in such a graph represent the points in which the response reaches equal values (iso-response curves). Figure 1a clearly shows that Cd accumulation increases only along the abscissa, from left to right (0.8–3.8 mg kg^−1^), which means from lower to higher concentrations of Cd, with only a slight deviation on the vertical axis due to the weak interaction with Cr. Moreover, the calculated semiamplitudes of the 95% confidence intervals (Figure 1b) show that the region in which the response is significantly different from zero (at α = 0.05) is the right part of the response surface, at high levels of “Cd”. This is a confirmation of the fact that *P. aviculare* can be considered a good bioindicator for Cd because the concentration in the plant depends only on the concentration in the medium without any interferences from other metals. The proportional uptake of Cd has been demonstrated in this species also by Salinitro et al., 2020 [28], who found increasing levels of this metal in plant shoots at increasing concentrations in the nutrient solution. Although Cd is not an essential nutrient for plants, it is easily absorbed by the roots, in which it follows the same cellular pathways used by Zn, which is an essential micronutrient [29].

### 3.2. Chromium

For the present study, Cr(III) was used instead of the more toxic Cr(VI) [30] because it is known that Cr(VI) interferes with the photosynthetic pathways causing serious damage to the plant growth. In the present study the focus was to study whether Cr interacted with other metals, rather than its effects on the plant. It is known that Cr is absorbed by non-specific channels, unlike other essential ions that are strictly regulated [31], therefore its uptake is concentration dependent.

The plant concentrations obtained for Cr range from 0.06 to 3.5 mg kg^−1^ (Table A1). The Cr accumulation by *P. aviculare* is mostly affected by its concentration in the medium. It is confirmed by the significance of the corresponding coefficient (*b*_3_, *p* < 0.001). Another less statistically significant factor for Cr is the square effect of Pb (*b*_22_, *p* = 0.0354), but the confidence interval of its coefficient is very close to zero.

**Figure 2:**
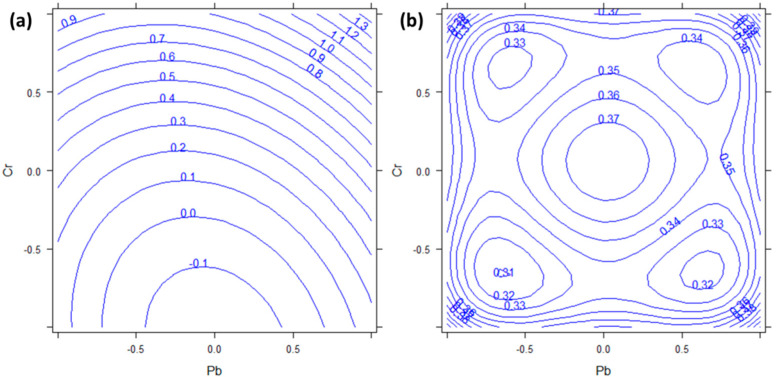
(**a**) Response surface and (**b**) semiamplitude of the 95% confidence interval for Cr response. Factors Cr and Pb are reported in abscissa and ordinate respectively. Cd has been kept constant to the 0 level.

The response surface reported in Figure 2 shows the absorption of chromium calculated by the DoE model as a function of the factors Cr and Pb. From Figure 2a, it can be seen that the Cr concentration in the plant increases almost linearly with the concentration of Cr in the medium, with only a slight curvature due to the effect of Pb. Moreover, the semiamplitdes of the 95% confidence intervals in Figure 2b indicate that only the region at high levels (+1) of Cr shows response values significantly different from zero. These results indicate that *P. aviculare* can be a good candidate for Cr bioindication. However, it has to be taken into account that, under field conditions, the bioavailability of Cr is generally low due to its strong adsorption by organic matter [29] and in our hydroponic conditions we were not able to evaluate such a drawback.

### 3.3. Lead

**Figure 3:**
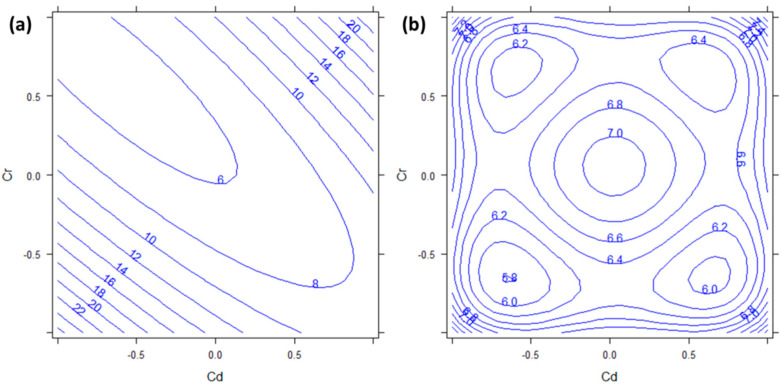
(**a**) Response surface and (**b**) semiamplitude of the 95% confidence interval for Pb response. Factors Cd and Cr are reported in abscissa and ordinate respectively. Pb has been kept constant to the 0 level.

The concentration range obtained for Pb goes from 0.56 to 49 mg kg^−1^ (Table A1). The DoE model for Pb indicates a strong significance of the interaction between Cd and Cr in its absorption (*b*_13_, *p* < 0.001) and a lesser significance of the Cr squared effect (*b*_22_, *p* = 0.482). Interestingly, the Pb factor is not significant neither by itself nor as interaction or squared effect. Figure 3 shows the response surface of Pb as a function of the factors Cd and Cr. From Figure 3a, it can be seen that the highest concentration of Pb can be reached when both Cd and Cr are at the lowest or at the highest levels. The semiamplitudes of the 95% confidence intervals (Figure 3b) indicate that these regions are also the only ones with response values significantly different from zero. Considering that most Pb salts are insoluble, this ion may have reacted with the other ions available in the nutrient solution making insoluble complexes not available to plants. Pb, indeed, could precipitate as PbSO_4_ or Pb_3_(PO_4_)_2_. The low availability of Pb has been reported also in heavily polluted soil, where, with concentrations of 850 mg kg^−1^, the uptake in the plant was very low (125 mg kg^−1^ in shoots) [32]. In light of the present results, we can conclude that *P. aviculare* is not a good candidate to be used for Pb bioindication. Further studies are needed to understand whether this non-linear uptake of lead was due to physiological characteristic intrinsic in the species or unavailability of this element in the growing medium. It would also be interesting to evaluate if the presence of Cd and Cr really affects the Pb absorption once the availability of Pb in the growing medium is ascertained.

### 3.4. Copper

Copper, together with Zinc, was kept at a fixed concentration (0.5 and 2.0 μM respectively) in all the experiments of DoE. We decided not to vary these elements because they are also essential nutrients for plants if present at low levels. In the present study these two elements were kept constant not to cause metal stress in plants, therefore only the influence that other metals had on the absorption of these two was evaluated. The plant concentrations obtained for Cu ranged from 3.5 to 29 mg kg^−1^ (Table A1).

**Figure 4:**
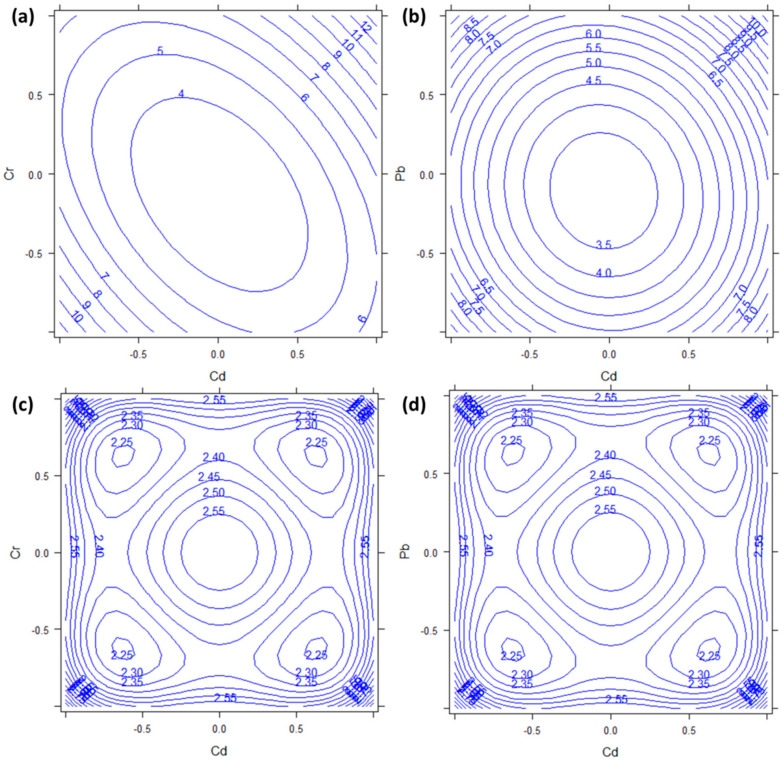
Response surface and semiaplitudes of the 95%confidence intervals for Cu response: (**a**) response surface with Cd and Cr in abscissa and ordinate; (**b**) response surface with Cd and Pb in abscissa and ordinate; (**c**) semiaplitude of the 95% confidence interval with Cd and Cr in abscissa and ordinate; (**d**) semiaplitude of the 95% confidence interval with Cd and Pb in abscissa and ordinate. The third factor, not represented in each plot, has always been kept constant to the 0 level.

Regarding Cu, Table 2 shows that none of the factor metals (Cd, Cr, and Pb) was significant by itself. However, all the square effects (*b*_11_, *b*_22_, and *b*_33_, *p*_11_ = 0.001 and *p*_22_ = 0.0044, *p*_33_ = 0.0077) and the interaction between Cd and Cr (*b*_13_, *p* < 0.001) were significant. Figure 4, therefore, shows the response surfaces of Cu concentration as a function of Cr vs. Cd and Pb vs. Cd. Figure 4a,b shows the minima of absorption for Cu (with response values not significantly different from zero, due to the semiamplitudes of the 95% confidence intervals reported in Figure 4c,d) when both Cd, Cr, and Pb were at the urban (0) levels. The absolute maxima (at least for the explored space) were found when both Cd and Cr were at the highest or at the lowest level (Figure 1a) and for both high and low levels of Pb (Figure 2b). The strong influence of the other ions on Cu uptake makes the bioindication of this element not feasible with *P. aviculare.* Overall, the literature agrees that the absorption of Cu by plants is strictly regulated, therefore the level of this element is maintained constant within plant tissue for a broad range of Cu concentrations in the growing medium [29,33]. For the previous reasons, we did not find any definitive explanation for such behavior of Cu, and more studies are needed.

### 3.5. Zinc

**Figure 5:**
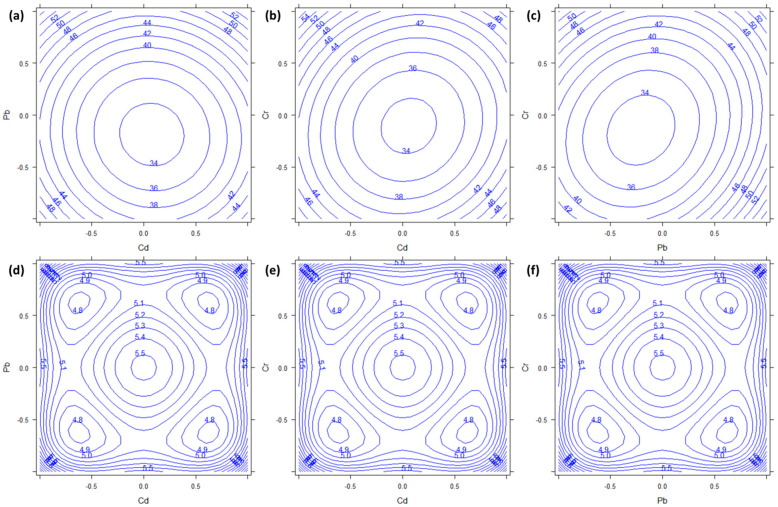
Response surfaces and semiamplitudes of the 95% confidence intervals for Zn dependent variable. Factors are: (**a**,**d**) Pb vs. Cd; (**b**,**e**) Cd vs. Cr; (**c**,**f**) Pb vs. Cr. The third factor, not represented in each plot, has always been kept constant to the 0 level.

The plant concentrations obtained for Zn ranged from 32 to 81 mg kg^−1^ (Table A1). Furthermore, in the case of Zn, Table 2 shows a strong significance of the square effects (*b*_11_, *b*_22_, and *b*_33_; all *p* < 0.001). In this case, there was also a significant effect of Pb (*b*_2_, *p* = 0.0089) and of the interaction between Pb and Cr (*b*_23_, *p* = 0.0306). Figure 5, therefore, shows all the combinations of the three factors. In all cases, the absorption of Zn showed minima in the central (urban) levels of all factors, while it increased in the corners of the response surfaces. In particular, both Figure 5a,c show a local maximum when the concentration of Pb was highest. Moreover, considering the semiamplitudes of the 95% confidence intervals reported in Figure 5d–f, all the calculated response values were significantly different from zero. In this case, we did not find any explanation for such behavior of Zn. It seems clear that its accumulation is strongly dependent on the presence of the other metals, therefore the *P. aviculare* would not be useful for the bioindication of Zn.

## 4. Conclusions

A design of experiment in full factorial mode, with three factors varied over three levels, has been applied to study the metal bioindication potential of *P. aviculare* for the elements Cd, Cr, Cu, Pb, and Zn and their interactions in their uptake.

Some significant effects of interaction between the factors (metals) have been observed, making it possible to model the plant uptake of a certain element during the growth in polymetallic substrates (in this study, the hydroponic nutrient solution). This preliminary study has demonstrated that the DoE is a useful tool to forecast (or validate) the bioindication potential of plants species before their application at field levels.

The present study showed that *P. aviculare* can be effectively used as a bioindicator of Cd and Cr, since the models indicated the absence of interactions with the other metals and a dose-dependent linear uptake of these elements (with *p*-values < 0.001). On the other hand, this species is unreliable for the bioindication of Pb, Cu, and Zn whose uptake was strictly influenced by the presence of all the other metals in the medium. It was also shown that, in general, the uptake of a certain metal cannot be considered independently from the presence of other metals in the field, but attention must be paid to the interaction of the metal of interest with the others. However, this kind of study must be accompanied by physiological investigation related to the metal uptake mechanisms, as well as by field trials useful to confirm the actual bioindication effectiveness of the element selected through the DoE. Moreover, other variables such as a soil’s physicochemical characteristics must be included in the model to make it more realistic and similar to actual environmental conditions. In the future, more studies are needed that include other plant species and variables to confirm the feasibility of this approach for the selection of bioindicator species.

## Figures and Tables

**Table 1:** Metals concentrations corresponding to countryside (low), urban (central) and heavily polluted soil (high) utilized for the DoE.

Level	Low	Central	High
Concentration	μM	μM	μM
Cd	0.0100	0.0700	0.140
Pb	1.83	14.5	29.0
Cr	6.92	23.1	46.2

**Table 2:** Regression coefficients estimated for the models of DoE. *b*_0_ is the intercept, the other subscripts of the regression coefficients (*b_i_*) indicate the *i*-th factors (metals): (1) Cd; (2) Pb; (3) Cr. Bold values indicate the statistically significant factors (*p*-values lower than α = 0.05), stars indicate the significance level (* indicates *p* < 0.05; ** *p* < 0.01; *** *p* < 0.001).

Model	Cd	Cr	Pb	Cu	Zn
*b*_0_	3.1	0.14	5.9	3.2	33
*b*_1_	0.87 ***	−0.081	0.22	0.49	−1.4
*b*_2_	0.12	0.12	1.6	0.65	3.4 **
*b*_3_	−0.0073	0.53 ***	−1.7	0.82	1.9
*b*_12_	−0.050	−0.12	−3.6	0.49	0.64
*b*_13_	−0.58 *	−0.046	8.9 ***	2.9 ***	−2.0
*b*_23_	−0.30	0.12	0.20	−0.80	−3.4 *
*b*_11_	−0.32	0.23	4.6	3.6 **	9.5 ***
*b*_22_	−0.043	0.32 *	−0.65	3.1 **	9.7 ***
*b*_33_	−0.55	0.23	5.8 *	3.0 **	9.3 ***

## Data Availability

The data are available from the authors under request.

## Sample Availability

Samples are not available.

## Appendix A

**Table A1:** DoE table. Factor levels are the ones reported in Table 1. Cu and Zn had fixed concentrations (0.5 and 2.0 μM, respectively). Concentrations are expressed in mg kg^−1^ (metal weight over plant weight).

	Factors (X) Block			Responses (Y) Block				
	Cd	Pb	Cr	Cd_plant_	Cr_plant_	Pb_plant_	Cu_plant_	Zn_plant_
Sample 1_1	−1	−1	−1	0.53	0.325	3.48	10.6	63.4
Sample 1_2	−1	−1	−1	0.55	0.342	3.43	10.6	67.8
Sample 1_3	−1	−1	−1	0.56	0.306	3.35	10.3	63.2
Sample 2_1	−0.08	−1	−1	0.88	0.138	25.1	8.23	40.3
Sample 2_2	−0.08	−1	−1	0.85	0.120	44.9	8.06	41.6
Sample 2_3	−0.08	−1	−1	0.82	0.121	36.9	8.06	42.0
Sample 3_1	1	−1	−1	2.09	0.122	3.69	9.43	59.8
Sample 3_2	1	−1	−1	2.35	0.155	3.53	9.72	58.7
Sample 3_3	1	−1	−1	2.34	0.144	3.86	9.11	57.7
Sample 4_1	−1	−0.07	−1	0.66	0.185	48.8	7.53	38.5
Sample 4_2	−1	−0.07	−1	0.66	0.171	45.4	7.71	37.5
Sample 4_3	−1	−0.07	−1	0.64	0.160	46.3	5.24	38.4
Sample 5_1	−0.08	−0.07	−1	3.26	0.146	3.87	7.28	46.4
Sample 5_2	−0.08	−0.07	−1	3.25	0.133	3.78	6.75	47.2
Sample 5_3	−0.08	−0.07	−1	3.18	0.146	3.80	7.72	46.7
Sample 6_1	1	−0.07	−1	3.36	0.194	2.03	8.28	36.4
Sample 6_2	1	−0.07	−1	3.34	0.178	2.03	9.50	37.1
Sample 6_3	1	−0.07	−1	3.31	0.165	2.11	8.11	37.3
Sample 7_1	−1	1	−1	0.55	0.263	29.8	21.1	69.5
Sample 7_2	−1	1	−1	0.56	0.296	27.5	22.7	74.9
Sample 7_3	−1	1	−1	0.53	0.288	30.4	22.6	62.7
Sample 8_1	−0.08	1	−1	2.47	0.195	9.48	7.97	53.4
Sample 8_2	−0.08	1	−1	2.49	0.163	9.81	8.24	53.4
Sample 8_3	−0.08	1	−1	2.54	0.175	10.1	8.24	52.6
Sample 9_1	1	1	−1	6.54	0.297	10.8	7.13	72.3
Sample 9_2	1	1	−1	6.92	0.297	11.5	7.63	73.7
Sample 9_3	1	1	−1	6.25	0.312	11.8	7.85	73.0
Sample 10_1	−1	−1	−0.18	2.29	0.599	3.10	6.45	42.1
Sample 10_2	−1	−1	−0.18	2.33	0.574	4.98	7.07	39.8
Sample 10_3	−1	−1	−0.18	2.28	0.576	4.98	7.43	40.7
Sample 11_1	−0.08	−1	−0.18	4.66	0.222	1.56	6.20	37.3
Sample 11_2	−0.08	−1	−0.18	4.64	0.210	1.64	6.38	35.9
Sample 11_3	−0.08	−1	−0.18	4.51	0.204	1.68	6.05	35.9
Sample 12_1	1	−1	−0.18	4.83	0.067	0.78	6.86	42.6
Sample 12_2	1	−1	−0.18	4.83	0.057	0.63	6.93	42.1
Sample 12_3	1	−1	−0.18	4.90	0.060	0.56	7.60	41.7
Sample 13_1	−1	−0.07	−0.18	0.35	0.260	12.4	7.97	38.1
Sample 13_2	−1	−0.07	−0.18	0.34	0.248	12.2	7.18	38.4
Sample 13_3	−1	−0.07	−0.18	0.34	0.251	12.3	7.58	39.3
Sample 14_1	−0.08	−0.07	−0.18	5.16	0.964	15.8	9.32	48.4
Sample 14_2	−0.08	−0.07	−0.18	5.48	1.01	16.5	9.47	49.0
Sample 14_3	−0.08	−0.07	−0.18	5.21	0.994	16.7	9.54	49.0
Sample 15_1	1	−0.07	−0.18	1.13	0.279	25.1	7.80	49.1
Sample 15_2	1	−0.07	−0.18	1.11	0.291	24.3	7.29	49.0
Sample 15_3	1	−0.07	−0.18	1.11	0.281	24.1	7.54	48.4
Sample 16_1	−1	1	−0.18	2.86	0.161	4.64	8.67	57.4
Sample 16_2	−1	1	−0.18	2.85	0.156	4.64	8.75	57.0
Sample 16_3	−1	1	−0.18	2.93	0.124	4.91	8.75	55.7
Sample 17_1	−0.08	1	−0.18	1.49	0.944	9.35	7.13	52.4
Sample 17_2	−0.08	1	−0.18	1.45	0.954	8.17	7.13	52.7
Sample 17_3	−0.08	1	−0.18	1.46	0.935	8.17	5.38	52.7
Sample 18_1	1	1	−0.18	2.49	0.310	7.90	8.18	48.8
Sample 18_2	1	1	−0.18	2.49	0.305	8.07	8.22	58.8
Sample 18_3	1	1	−0.18	2.71	0.294	8.58	8.34	46.8
Sample 19_1	−1	−1	1	0.62	1.72	1.57	16.1	81.1
Sample 19_2	−1	−1	1	0.65	1.62	1.53	15.4	78.8
Sample 19_3	−1	−1	1	0.63	1.59	1.35	16.1	77.5
Sample 20_1	−0.08	−1	1	1.41	0.241	2.24	7.53	33.7
Sample 20_2	−0.08	−1	1	1.43	0.239	2.48	7.69	34.9
Sample 20_3	−0.08	−1	1	1.46	0.352	2.35	7.65	32.0
Sample 21_1	1	−1	1	4.63	2.10	36.3	18.1	74.1
Sample 21_2	1	−1	1	2.96	2.33	42.8	17.7	73.9
Sample 21_3	1	−1	1	2.61	2.30	48.5	17.4	69.2
Sample 22_1	−1	−0.07	1	3.46	0.187	3.12	8.00	66.5
Sample 22_2	−1	−0.07	1	3.58	0.171	3.04	7.74	64.6
Sample 22_3	−1	−0.07	1	3.40	0.173	2.88	7.93	65.6
Sample 23_1	−0.08	−0.07	1	1.91	1.17	3.48	7.75	43.3
Sample 23_2	−0.08	−0.07	1	1.86	1.18	3.56	9.07	43.0
Sample 23_3	−0.08	−0.07	1	1.83	1.16	3.65	6.52	43.6
Sample 24_1	1	−0.07	1	3.11	0.363	3.60	4.57	49.5
Sample 24_2	1	−0.07	1	3.09	0.360	3.66	4.97	49.5
Sample 24_3	1	−0.07	1	3.15	0.356	3.73	4.86	39.7
Sample 25_1	−1	1	1	1.93	3.39	24.1	7.74	59.2
Sample 25_2	−1	1	1	1.97	3.44	25.2	8.05	58.7
Sample 25_3	−1	1	1	2.01	3.48	20.6	8.06	59.2
Sample 26_1	−0.08	1	1	2.47	0.321	3.51	3.58	60.4
Sample 26_2	−0.08	1	1	2.52	0.328	3.80	3.54	60.8
Sample 26_3	−0.08	1	1	2.40	0.326	3.94	3.83	60.8
Sample 27_1	1	1	1	2.65	1.76	28.4	28.9	60.8
Sample 27_2	1	1	1	2.40	1.79	28.6	28.8	58.2
Sample 27_3	1	1	1	2.18	1.82	26.4	29.1	57.7
